# Multivisceral Resection in Robotic Liver Surgery

**DOI:** 10.3390/cancers14020355

**Published:** 2022-01-12

**Authors:** Kevin M. Sullivan, Yuman Fong

**Affiliations:** Department of Surgery, City of Hope National Medical Center, Duarte, CA 91010, USA; kesullivan@coh.org

**Keywords:** minimally invasive surgery, robotic, liver surgery, liver resection, hepatectomy

## Abstract

**Simple Summary:**

Liver surgery can be performed simultaneously with operations to remove other organs in certain circumstances, such as removal of colorectal cancer in the colon or rectum at the same time as metastatic lesions to the liver. These types of operations have been performed as open or laparoscopic procedures; however, more recently, they can be performed with a robotic approach. In this article, we review the literature and describe robotic liver resections performed with robotic resection of other organs, including colon, rectum, and pancreas. These published reports demonstrate that, in select cases and experienced hands, robotic multivisceral resection can be safely performed with good outcomes.

**Abstract:**

Minimally invasive surgery techniques are expanding in utilization in liver resections and now include robotic approaches. Robotic liver resection has been demonstrated to have several benefits, including surgeon ergonomics, wrist articulation, and 3D visualization. Similarly, for multivisceral liver resections, the use of minimally invasive techniques has evolved and expanded from laparoscopy to robotics. The aim of this article is to review the literature and describe multivisceral resections, including hepatectomy, using a robotic technique. We describe over 50 published cases of simultaneous robotic liver resection with colon or rectal resection. In addition, we describe several pancreatectomies performed with liver resection and one extra-abdominal pulmonary resection with liver resection. In total, these select reported cases at experienced centers demonstrate the safety of robotic multivisceral resection in liver surgery with acceptable morbidity and rare conversion to open surgery. As robotic technology advances and experience with robotic techniques grows, robotic multivisceral resection in liver surgery should continue to be investigated in future studies.

## 1. Introduction

With the wide adoption of minimally invasive surgery (MIS) for various operations, the utilization of laparoscopic and, eventually, robotic approaches has steadily increased in liver surgery. Compared to open liver surgery, MIS approaches have been demonstrated to have a faster recovery time with fewer short-term complications [1,2]. Importantly, these advantages in recovery over open surgery are not gained at the expense of oncologic outcomes, which remain similar for robotic surgery of both primary and metastatic liver tumors [3]. While initial perioperative costs are greater for robotic surgery, the total cost for robotic surgery is similar or decreased compared to open surgery [4,5]. When comparing MIS approaches, robotic liver surgery has been demonstrated as feasible and safe compared to laparoscopy [6]. Long-term oncological outcomes, including overall and disease-free survival for primary hepatobiliary malignancies and metastatic colorectal cancer (CRC), are comparable when a robotic approach is used versus both open and laparoscopic techniques [7,8]. While maintaining similar benefits of reduced hospital length of stay and short-term complications to laparoscopy as an MIS technique, notably, robotic surgery has several advantages over laparoscopy including surgeon ergonomics, three-dimensional visualization with the robotic camera, and the ability to articulate wrists using robotic equipment. Additionally, the posterosuperior segments 4A, 7, and 8 are technically challenging to access via laparoscopy, but resection in these segments is feasible using robotic surgery [9]. Building on the experience of laparoscopic minimally invasive multivisceral resection as the learning curve for robotic surgery, experienced centers have expanded minimally invasive multivisceral resections to include a robotic approach. In this article, we review the use of robotic surgery to perform such multivisceral liver resections.

## 2. Combined Hepatic and Colorectal Resection for Synchronous Colorectal Liver Metastases

A common indication for hepatectomy is CRC metastatic to the liver. Once considered only for palliative chemotherapy, patients with CRC liver metastases (CRCLM) undergoing resection of metastatic lesions now experience a 5-year survival of about 25–51% and a 10-year survival of 17–36% [10]. Many of the patients treated by hepatectomy are cured.

Simultaneous open operative resection of synchronous CRCLM with a primary CRC tumor has been demonstrated to be safe and feasible in select patients and have favorable outcomes than staged operations [11,12]. Analysis of the LiverMetSurvey demonstrated that simultaneous resection of liver metastases and the CRC primary tumor has similar morbidity and survival for low-complexity hepatic resections, while a liver-first approach had a survival advantage only when the hepatic metastases were multiple and bilobar [11]. A randomized trial (METASYNC) of simultaneous versus delayed resection of liver metastases after resection of CRC showed improved 2-year survival in the simultaneous resection group without statistically significant differences in complication rate [12]. Synchronous colorectal and liver resection has also been demonstrated to be safe and feasible in well-selected patients via a laparoscopic MIS approach [13].

Reports of robotic simultaneous colon and liver resection are beginning to be published. The first published case of a simultaneous robotic colorectal and liver resection was in 2008 [14]. The total operative time was 360 min without additional port placement. In 2017, Sunil et al. [15] published a case report of simultaneous robotic-assisted resection of rectosigmoid cancer located 12 cm from the anal verge and a 2.1 cm segment 4A/8 liver metastasis. The operation was performed with a single re-docking of the robot with an operative time of 390 min and an estimated blood loss (EBL) of 300 mL. The patient’s length of stay (LOS) was 6 days, and the final pathology revealed moderately differentiated adenocarcinoma with 2/36 positive lymph nodes and negative margins of both the colorectal and liver specimens. This patient had not undergone neoadjuvant chemotherapy and instead was treated with adjuvant systemic therapy, with follow-up at 20 months without recurrence. Morelli et al. [16] also reported a case series of three patients who underwent combined anterior rectal resection and hepatic resection, with operative time ranging from 360–480 min, each completed with two dockings and a median EBL of 200 mL. Eu et al. [17] published an experience of da Vinci Xi combined robotic low anterior resection and resection of a solitary segment 2/3 liver metastasis using two 8 mm ports in the right abdomen, one periumbilical 8 mm port, and one 8 mm port in the left upper quadrant, as well as a 12 mm assist port in the left abdomen. Dwyer et al. [18] reported an additional case series of six CRC metastatic to the liver with no conversions to laparotomy, a mean operative time of 401 min, EBL of 316 mL, and a hospital stay of 4.5 days. There was one anastomotic leak in the series and two pelvic abscesses, but no 30-day mortalities. In rectal cancer, Soh et al. [19] reported 13 patients who underwent robotic rectal resection with an additional procedure, 4 of which were robotic hepatobiliary procedures. There was no reported difference in LOS of postoperative complications, including anastomotic leak or bleeding compared to rectal-only procedures.

In 2019, the largest case series to date was published by Navarro et al. [20] and included six wedge hepatectomies, one caudate lobectomy, two right hepatectomies, one left hepatectomy, one left lateral segmentectomy, and one associating liver partition and portal vein ligation for staged hepatectomy (ALPPS) procedure. In this series, mean operative time was 449 min with mean EBL 274.3 mL. There were no conversions to laparotomy, with a single grade I complication, one grade II complications, and two grade III complications, including one anastomotic leak and two liver abscesses. Giovanetti et al. [21] report an additional series of five patients undergoing robotic combined robotic and colorectal resection with complications of one ICU admission, one umbilical cellulitis, and one ileus, but no 30-day mortality. Similarly, Masetti et al. [22] report a fully robotic ALPPS with simultaneous left colectomy for synchronous CRCLM. Additionally, our group has reported three cases of robotic multivisceral resection, including one combined colorectal (right hemicolectomy) and liver resection (partial segments 5/6) whose postoperative course was complicated by ileus. Pathology, in this case, was notable for negative margins with 17 negative lymph nodes, and the patient had no readmissions after 13 months of follow-up [23].

A review of eight studies collectively reported no conversions to open with combined robotic-assisted colorectal and liver resection, a 28.6% rate of total postoperative complications, and one (3.6%) postoperative mortality in a patient who died of CRC [24]. Most recently, the experience of 28 cases from the Minimally Invasive Robotic-Assisted combined Colorectal and Liver Excision Surgery (MIRACLES) study was reported. In this study, operative time was 332 min, EBL 143 mL, and LOS 8 days. Two conversions to laparotomy were reported, and three grade III–IV complications were noted. There were no mortalities. Oncologic outcomes included one positive R1 margin, and median OS was 27.5 months. Overall, the published data (summarized in Table 1) on combined colorectal and liver resections using robotic techniques demonstrate an acceptable operative time and EBL with minimal severe complications and a low mortality rate.

## 3. Combined Hepatic and Other Abdominal Solid Organ Resection

With growing experience and adoption of robotic distal pancreatectomy, several authors have published combined pancreatectomy and hepatectomy. Calin et al. [26] published the first case report of a combined robotic distal pancreatectomy and hepatectomy for neuroendocrine tumor (NET) metastatic to the liver. The operative time for this combined procedure was 369 min with an EBL of 100 mL. In 2020, Bhat et al. [27] also reported a robotic distal pancreatectomy, splenectomy, sleeve gastrectomy, cholecystectomy, and resection plus ablation of multiple liver lesions for well-differentiated NET with a 4-day hospital LOS. Villano et al. [28] also report a patient with acinar cell carcinoma of the pancreas who underwent neoadjuvant chemotherapy followed by robotic distal pancreatectomy, splenectomy, and non-anatomic resection of segment 6. The LOS following the operation was 2 days with no complications, and pathology revealed negative margins. Our group has performed a combined distal pancreatectomy, splenectomy, and partial segment 3 resection for pancreatic ductal adenocarcinoma [23]. The pathology from this tumor was pT2N0 with negative margins, 12 negative lymph nodes, and no viable cancer in the liver lesion. The patient had no readmissions or other complications at 13 months of follow-up.

In addition to pancreatectomy, our group has performed multivisceral resection, which may be indicated for other primary cancer, such as renal cell carcinoma (RCC). We have also reported a combined robotic debulking and resection of multiple intraabdominal and retroperitoneal masses with a segment seven resection and cholecystectomy for recurrent, metastatic RCC [17]. The patient’s pathology showed three RCC metastases (1–3.2 cm) resected with negative margins, three negative lymph nodes, and chronic cholecystitis. The patient also had no readmissions or other complications at 23 months of follow-up. These cases illustrate the feasibility of multivisceral robotic resection of pancreatic or other intraabdominal resections in combination with hepatic resection.

Table 2 displays a comparative summary of simultaneous robotic liver resection and other abdominal resection. 

## 4. Combined Hepatic and Extra-Abdominal Resection

To date, there is one published case of a hepatic resection combined with an extra-abdominal thoracic resection. Xu et al. [29] report a 59-year-old man diagnosed with a cT3N0 rectal adenocarcinoma with solitary liver and lung metastases who underwent combined lung, liver, and rectal resections using robotic approaches for each. In this case, a right lower lobe wedge resection of the lung was carried out first, followed by repositioning the patient supine and segmental hepatectomy of the liver metastasis. Finally, the patient was placed in a modified lithotomy position. Anterior resection of the rectum with a distal staple line of 5 cm from the anal verge and a primary anastomosis were performed. The lung resection was performed in 30 min, the liver resection in 270 min, and the rectal resection in 90 min; in total, the procedure was completed in 480 min when including docking time of 90 min in addition to the console time of 390 min. EBL was 600 mL, and the patient had an uneventful postoperative course with LOS of 7 days. The final pathology was pT3N1M1, with all margins negative. In a highly selected patient, this case report illustrates the ability to perform three simultaneous operations as a one-stage procedure with a short overall recovery time, with the limitation of prolonged operative time for multiple instances of repositioning and re-docking the robot.

## 5. Conclusions

**Figure 1:**
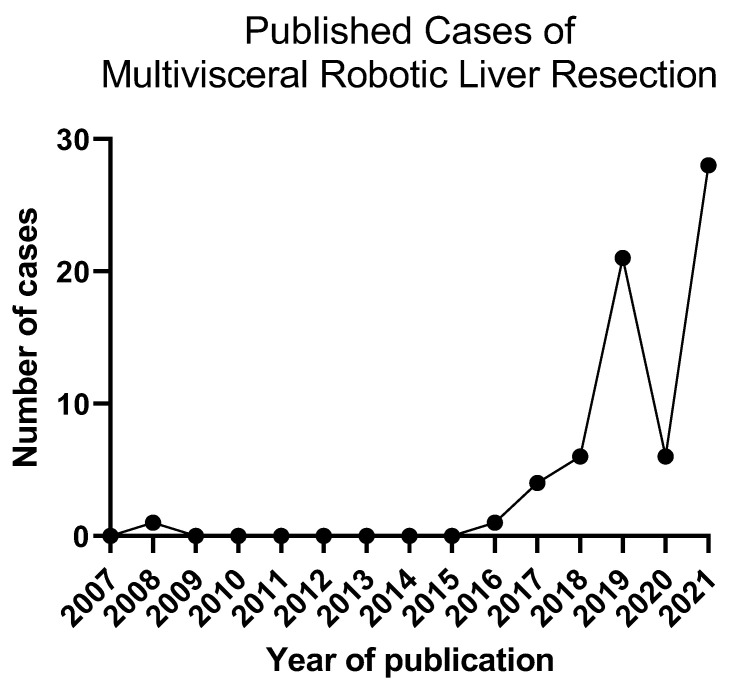
Trend of published cases of multivisceral robotic resection over time.

In this article, we have reviewed the reported cases of simultaneous robotic multivisceral resection in liver surgery, including combined colectomy or proctectomy, pancreatectomy, and other resections. With combined robotic colorectal and hepatic resection, a systematic review of 50 cases has been reported in the literature thus far, with a single death due to the underlying cancer at 26 months [24]. Over time, the number of published cases per year has shown an increasing trend (Figure 1). The lack of any reported 30-day mortality and acceptable complication rate demonstrates the feasibility of this approach in select cases. Similarly, several cases of combined robotic distal pancreatectomy with hepatectomy have been published with short LOS and minimal complications. Given these positive early findings as well as a shorter learning curve for robotic surgery compared to laparoscopic surgery [30], the number of high volume robotic centers, and therefore the adoption of robotic multivisceral resection, will likely expand.

Overall, these published reports of multivisceral robotic liver surgery likely represent highly selected patients and may represent a publication bias of positive outcomes. Nonetheless, robotic surgery does offer advantages in the ability to perform multivisceral resection via a minimally invasive approach. Such simultaneous procedures also allow other advantages of robotic surgery, including assessing perfusion by indocyanine green (ICG) and reducing wound infection or incisional hernia. Although the use of ICG was not specifically described in most reports reviewed except for Konstantinidis et al., this technology is one example of a potential advantage of robotic surgery; amongst others, the technology continues to advance.

With careful planning, port location can be selected in such a manner as to use many of the same ports for both resections. During combined liver and CRC resections at our institution, we generally perform the liver resection first before proceeding to the colon resection. For this procedure, the patient was placed supine and adhesiolysis followed by intraoperative liver ultrasound, and then parenchymal transection was performed. Turning to the colon resection, the ileocolonic pedicle was sealed and divided, followed by medial to lateral mobilization of the ascending colon and then mobilization of the hepatic flexure. The colon and ileum were divided, and an intracorporeal anastomosis was performed with ICG assessment of the perfusion to the bowel. During our other experiences with non-colorectal primary tumors and liver metastases, the non-liver resections proceeded first. We positioned our patient for the combined liver–pancreas resection in the right lateral decubitus. After port placement and insufflation, the gastrocolic ligament was divided, as well as the short gastrics. The inferior pancreatic border was dissected, and the splenic artery divided, followed finally by pancreatic transection. Using the same ports, we transitioned to the liver resection, where intraoperative liver ultrasound followed by parenchymal transection was performed. Additionally, the robotic stapler was used, and therefore, a specialized bedside assistant was not required [23]. As technology advances, re-docking multiple times may no longer be necessary. Overall, the utilization of robotic approaches for multivisceral resection represents a new and innovative technical challenge in the surgical management of a complex multiorgan disease.

## Figures and Tables

**Table 1:** Summary of simultaneous robotic hepatic and colorectal resections for synchronous CRCLM.

Study	Cases	Liver Resection	Colorectal Resection	Operative Time (min)	EBL (mL)	LOS (Days)	Conversions	Complications	Other
Choi (2008) [14]	1	Segment 3	LAR	360	300	6	0	0	
Sunil (2017) [15]	1	Segment 4a/8	Recto-sigmoid	390	300	6	0	0	pT3N1bM1a
Morelli (2017) [16]	3	NR	AR	360–480	200	6	0	0	
Eu (2018) [17]	1	Segment 2/3	LAR	300	10	2	0	0	
Dwyer (2018) [18]	6	4 multiple Seg	3 LAR, 1 RC, 2 APR	401	316	4.5	0	3	1 anastomotic leak, 2 abscesses
Soh (2019) [19]	4	NR	NR	399	281	9.6	0	NR	
Navarro (2019) [20]	12	1 RH, 1 LH, 1 LLS, 1 CL, 1 ALPPS, 1 Seg, 5 wedge resections	7 LAR, 2 AR, 2 RC, 1 LC	449	274	NR	0	5	1 anastomotic leak, 2 abscesses
Giovannetti (2019) [21]	5	1 LLS, multiple Seg of 2, 3, 4, 6, 7	2 LAR, 2 RC, 1 APR	439	150	5	0	3	1 ICU, 1 cellulitis, 1 ileus; All margins negative
Masetti (2020) [22]	1	ALPPS	LC	NR	NR	NR	0	NR	
Konstantindis (2020) [23]	1	Segments 5 and 6 partial hepatectomies	RC	NR	50	8	0	Ileus	Negative margins, 0/17 positive LN
Ceccarelli (2021) [25]	28	20 wedge, 5 Seg, 1 LLS, 1 RH, 1 LH	9 RC, 7 LC, 10 LAR, 1 sigmoid, 1 APR	332	143	8	2	3 grade III-IV	1 R1 margin Median OS 27.5 months

NR, not reported; AR, anterior resection; LAR, low anterior resection; RC, right hemicolectomy; LC, left hemicolectomy; APR, abdominoperineal resection; ALPPS, associating liver partition and portal vein ligation for staged hepatectomy; RH, right hepatectomy, LH, left hepatectomy; Seg, segmentectomy; LLS, left lateral sectionectomy; CL, caudate lobectomy.

**Table 2:** Summary of simultaneous robotic hepatic and non-colorectal resections for synchronous CRCLM.

Study	Pathology	Liver Resection	Other Resection Performed	Operative Time (min)	EBL (mL)	LOS (Days)	Complications	Other
Calin (2016) [26]	NET	NR	DP, splenectomy	369	100	NR	NR	
Bhat (2020) [27]	NET	Non-anatomic resection 2 lesions, ablation of multiple lesions	DP, splenectomy, gastrectomy, cholecystectomy	420	400	4	None	R1 resection
Villano (2020) [28]	ACC	Non-anatomic segment 6	DP, splenectomy	NR	NR	2	None	Disease free at 6-month follow up
Konstantindis (2020) [23]	PDA	Partial segment 3	DP, splenectomy	NR	NR	5	None	Negative margins
Konstantindis (2020) [23]	RCC	Segment 7	Retroperitoneal mass resection	NR	NR	3	None	Negative margins, BMI 50.4

NR, not reported; NET, neuroendocrine tumor; ACC, acinar cell carcinoma; PDA, pancreatic ductal adenocarcinoma; DP, distal pancreatectomy.
